# Attacks on healthcare facilities as an indicator of violence against civilians in Syria: An exploratory analysis of open-source data

**DOI:** 10.1371/journal.pone.0217905

**Published:** 2019-06-10

**Authors:** Sayaka Ri, Alden H. Blair, Chang Jun Kim, Rohini J. Haar

**Affiliations:** 1 UCSF Institute for Global Health Sciences, San Francisco, California, United States of America; 2 Department of Mathematics, University of California, Berkeley, California, United States of America; 3 Division of Epidemiology, School of Public Health, University of California, Berkeley, California, United States of America; Harvard Medical School, UNITED STATES

## Abstract

**Background:**

Grasping the human cost of war requires comprehensive evaluation of multiple dimensions of conflict. While the number of civilian casualties is a frequently used indicator to evaluate intensity of violence in conflict, the inclusion of other indicators may provide a more complete understanding of how war impacts people and their communities. The Syrian conflict has been specifically marked by attacks against healthcare facilities, and the advancement of technology has provided an avenue for remote data analysis of conflict trends. This study aims to determine the feasibility of using publicly available, online data of attacks on healthcare facilities to better describe population-level violence in the Syrian Civil War.

**Methods:**

This study utilized publicly available datasets from the Violations Documentation Center (VDC) and Physicians for Human Rights (PHR) to compare trends in attacks on healthcare facilities and civilian casualties from March 2011 to November 2017 in the Syrian Civil War. We used descriptive statistics, bivariate tests and a multivariable hypothesis testing model to measure the association between the two indicators while adjusting for confounding variables.

**Results:**

We examined for associations between attacks on healthcare facilities and overall civilian casualties. In the adjusted regression model, each attack on a healthcare facility in the Syrian conflict corresponded to an estimated 260 reported civilian casualties in the same month (95% CI: 227 to 294). This model adjusted for population displacement (using number of registered refugees as a proxy). The May 2014 interaction term, used a transition point of early/late war based on political events during that time, illustrated that each healthcare facility attack after May 2014 corresponded to a statistically significant decrease of 228 civilian deaths. This suggests that although attacks on healthcare facilities continued to contribute to overall civilian deaths, the scale that this was happening was lower after May 2014.

**Conclusion:**

In the Syrian Civil War, our findings suggest that the inclusion of other humanitarian indicators, such as attacks on hospitals, may add granularity to traditional indicators of violence (e.g. such as civilian casualties) to develop a more nuanced understanding of the warring tactics used and violence against civilians in the Syrian conflict. This exploratory case study represents a novel approach to utilizing open-source data along with statistical analysis to interpret violence against civilians. Future research could benefit from analyzing attacks on healthcare facilities and other civilian infrastructure concurrently with civilian casualty data for further data-driven utilization of open-source data.

## Introduction

Understanding violent trends against civilians is important in informing relief efforts, advocating for foreign policy, and holding stakeholders accountable to violations of international humanitarian law (IHL). Despite its importance, measuring the scope of violence towards civilians remains challenging [1]. Although many indicators have been applied to measure conflict-associated violence, the total number of civilian casualties is commonly used as the standard index to evaluate the severity of war [2–4]. However, the methods used to count casualties are often unreliable in an ongoing conflict [5]. The breakdown of civil registries and absence of a centralized death registration system can result in an under-reporting of total casualties. It may also result in an over-reporting of identified casualties as databases may contain duplicate counts, or may include reports of non-conflict related fatalities [6]. Since accurate documentation of civilian deaths is difficult, challenges may arise from using casualties as the sole indicator of violence against civilians.

While a measure of civilian casualties may not be a reliable indicator for conflict violence by itself, attacks on social infrastructure can be used to complement our understanding of conflict dynamics. Despite explicit protections under International Humanitarian Law (IHL), civilian infrastructure and social institutions globally have been vulnerable to becoming ‘collateral damage’ in military campaigns as part of the fog of war and deliberately targeted [7,8]. Emergency research has described how healthcare systems, which provide essential services to communities, have recently been systematically attacked in conflicts worldwide [9,10]. An attack on a medical facility can restrict access to vital healthcare services, destroy social networks, and force population displacement [11]. Recent reports illustrate that violence against healthcare infrastructure is not only becoming more frequent, but is also increasingly targeted, deliberate, and systematic.

The ongoing Syrian Civil War is among the most striking examples of healthcare infrastructure directly targeted as a strategy of war [12]. Combatants, including the Syrian Armed Forces and Russian military, have been responsible for the bombing of hospitals, looting of ambulances, and execution of healthcare workers [13]. An analysis of attacks on healthcare facilities reveals a pattern of repeated targeting in opposition-controlled areas to restrict access to healthcare and engage in siege warfare [14,15]. Due to technological advancements such as satellite technology and social media, data on healthcare facility attacks in Syria have been well-documented by local field surveillance teams and NGOs [16].

Despite the rigorous documentation of healthcare facility attacks in the Syrian Civil War, there has been a lack of a consensus on the war’s casualty count. The discrepancy of total casualty counts from multiple organizations in Syria ranges from less than 150,000 deaths to greater than 500,000 deaths [17,18]. The Office of the United Nations High Commissioners for Human Rights (OHCHR) stopped publishing figures for the Syrian death toll in 2014 due to the lack of reliability and potential inaccuracy of the information presented. Varying and controversial death toll estimates are not limited to Syria but have been extensively discussed in Iraq and other conflicts [19]. However, the Syrian conflict is unique for occurring in the era of social media, which has not only provided an increased ability to record deaths in conflict settings, but has also raised the expectations of accuracy.

To evaluate the feasibility of using an unconventional indicator of violence, this paper compares trends of attacks on healthcare facilities and civilian casualties to gain a nuanced understanding of the violence against civilians in the Syrian Civil War.

## Methods

Several non-governmental and not-for-profit organizations maintain publicly available, online databases of statistics from the Syrian Civil War. Databases were identified through a broad, web-based search and were chosen based on the following inclusion criteria: (1) open-source and available publicly, (2) availability of monthly aggregated data, (3) a published and transparent methodology for how data were collected and presented, and (4) a multi-tier verification procedure before publishing data on incidents. Given the wide variability in civilian death counts, the source for the civilian casualty database was selected based on additional requirements: (1) differentiation of conflict related deaths by civilian and combatant status, (2) data reported from March 2011 to November 2017, and (3) a database that was updated at least monthly.

For included databases, we extracted: (1) the monthly number of civilian casualties, (2) the monthly number of attacks on healthcare facilities, and (3) the monthly number of registered Syrian refugees. The number of registered Syrian refugees was collected to determine whether the refugee exodus had a significant effect on the association between civilian casualties and healthcare facility attacks.

### Quantitative data analysis

After extracting the data, the research team utilized graphing functions on Excel (v15.34) to compare trends in civilian casualties and attacks on healthcare facilities over time to determine whether the pattern of violence would be consistent between the two indicators.

Descriptive statistics were calculated to assess overall distributions within the dataset. Bivariate testing explored the relationship between civilian casualties and healthcare facility attacks. Civilian casualties were used as the baseline indicator to determine the effect of corresponding conflict related variables including the number of refugees, months since the start of the conflict, and an effect modifying variable differentiating healthcare facility attacks pre- and post-May 2014. Statistical tests included the Kruskall-Wallis, Wilcoxon Rank-Sum, and Spearman’s tests due to the non-normal distribution of the outcome variables.

**Fig 1:**
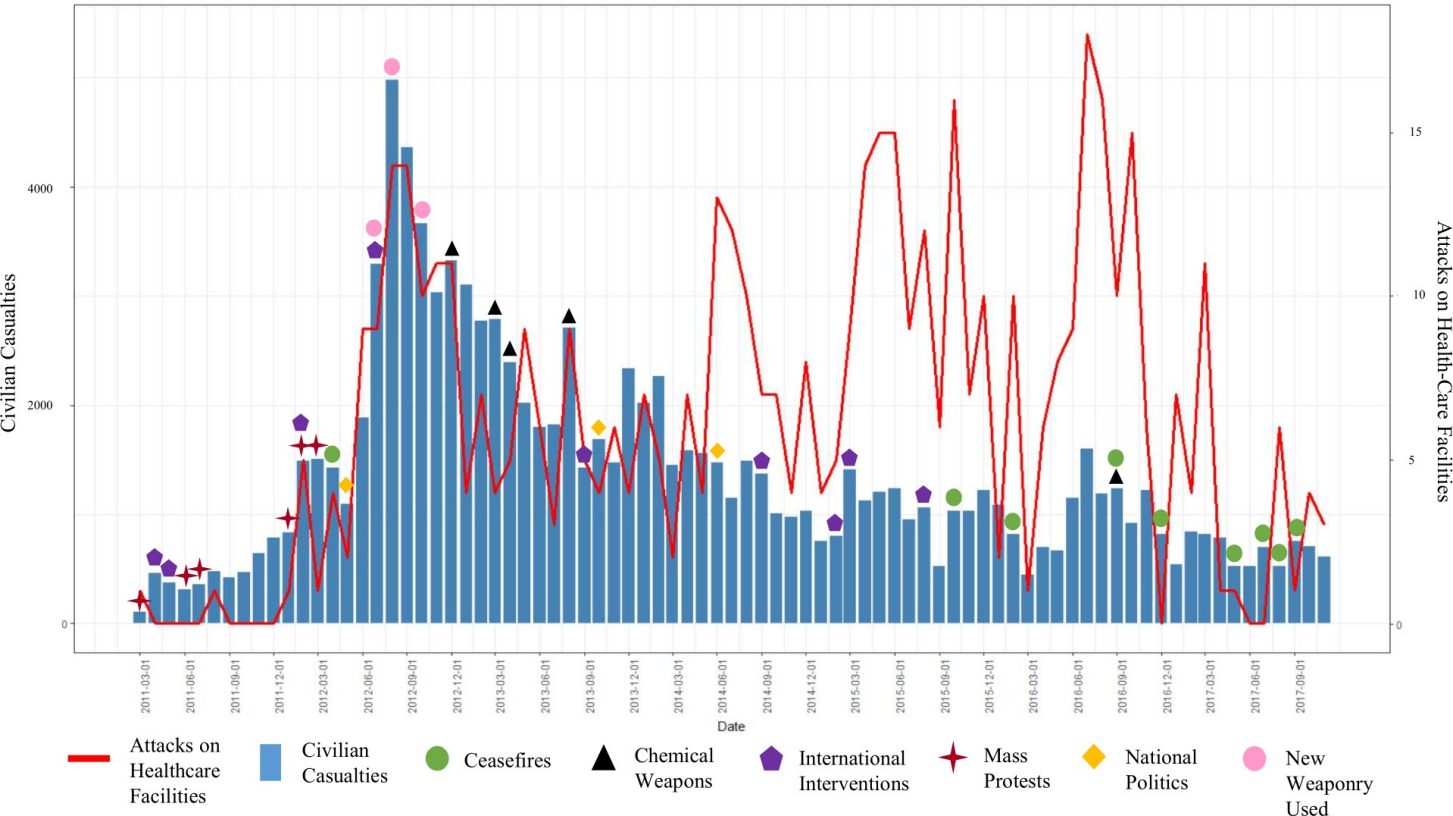
The Association of civilian casualties and attacks on healthcare facilities from March 2011 –November 2017 with significant events symbolized. Refer to Timeline of Significant Events in Supplementary Material (S2 Table).

A hypothesis testing multivariable linear regression model assessed the significance of the relationship between civilian casualties and attacks on healthcare facilities, in the presence of potential confounders. Due to the significant changes in the documented attacks on healthcare facilities and civilian casualties in the conflict after May 2014 in the bivariate analysis and visual analysis [Fig 1], we explored the potential for a pre-/post- effect modification. In holding with best practice, the component variables comprising the effect modification term were also included in the model. To assess the final model of best fit, nested models were compared using a partial F test, with the saturated models considered significantly better at a p<0.05 level. Unadjusted and adjusted point estimates, and 95% confidence intervals (95% CI) were calculated to show both the directionality and strength of associations. Estimates where the 95% CI did not cross zero were considered significant. All quantitative analyses were conducted with R Statistical Software V.3.3.3.

### Ethical considerations

There was no funding for this research and no ethics review was required for the use of open-source data in secondary data analysis.

## Results

Six online, publicly available databases that record information on the Syrian conflict were evaluated. These databases include Médecins Sans Frontières (MSF) and Physicians for Human Rights (PHR) for data on healthcare facility attacks, and the Syrian Center for Statistics and Research (CSR-SY), Syrian Observatory for Human Rights (SOHR), Syrian Network for Human Rights (SNHR), and the Violations Documentation Center (VDC) for civilian casualty data [20–25]. Based on the inclusion criteria discussed above, databases from PHR and the VDC were selected from this list (S1 Table). PHR provides the most comprehensive open-source dataset on healthcare facility attacks as the only organization that started documenting attacks from the start of the Syrian conflict in March 2011. VDC was chosen as the only organization to provide a database to distinguish between civilian or combatant casualties with a published, multi-tier methodology for users to review online. The UNHCR’s online database was also sourced to extract cumulative totals of registered Syrian refugees as a confounding variable (S1 Table). Table 1 outlines the sources, verification methodology, and descriptions of the online sources.

**Table 1:** Online sources for extracting monthly civilian casualties, monthly attacks on healthcare facilities, and monthly Syrian refugee counts.

Online Sources	Data Type	Duration of Documentation	Description of Documentation	Verification Methodology
**Violations Documentation Center (VDC)**^**[**39]^	Civilian Casualties	Mar. 2011 to present	1. Gather initial information from hospital registries, morgues, relatives or media sources 2. Confirm initial report with videos or photographs 3. Key information missing is investigated until published^44^	Three-stage verification process: initial reports, video/photo evidence, investigation until confirmed
**Physicians for Human Rights (PHR)**^**[**38]^	Attacks on Healthcare Facilities*	Mar. 2011 onwards	Uses social media, publications and field sources to identify attacks before publishing attacks on healthcare facilities and personnel on their online platform	Verification by at least two independent sources before publication of confirmed attacks
**United Nations High Commissioner for Refugees (UNHCR)**^**[**45]^	Registered Syrian Refugees	Mar. 2011 onwards	Government agencies, UNHCR field offices and NGOs contribute to UNHCR’s online database for cumulative refugee counts	Verification by three independent sources

*Attacks on healthcare facilities are defined as a violent assault (e.g. bombing, shelling, artillery, car bombs, shooting, arson, or attack by armed personnel) on a hospital, clinic, medical center, pharmacy, dispensary or field hospital.[21]

From March 2011 to November 2017, a total of 110,504 civilian casualties were reported with a median of 1364 civilian casualties per month. The range of civilian casualties varied greatly from 106 to 4,982 per month. During the same period, the number of healthcare facility attacks averaged 6.24 per month, ranging from 0 to 18 attacks per month.

Fig 1 illustrates a visual comparison of the pattern of violence against civilians by comparing monthly civilian casualty counts and attacks on healthcare facilities between March 2011 and November 2017. There are relatively similar trends in both civilian casualties and healthcare facility attacks from March 2011 to May 2014. This is most notable at violent peaks in August 2012 and August 2013. These months correlate with the use of new weaponry, which include the use of fighter jets and sarin nerve gas in Damascus (S2 Table). After May 2014, the intensity of violence recorded by civilian casualties and attacks on healthcare facilities appear to diverge. Civilian casualty counts stabilized to approximately 800 reported civilian casualties per month. Healthcare facility attacks varied more widely from zero to over 15 attacks per month. The deadliest period indicated by civilian casualties was from July to October 2012 when new Russian made cluster bombs and chemical gases were introduced (S2 Table). The highest number of healthcare facilities attack occurred from May 2015 to July 2016. Both monthly civilian casualties and healthcare attacks indicated a decrease in civilian violence from November 2016 to November 2017 due to a number of ceasefire attempts by local and international armed actors (S2 Table).

**Fig 2:**
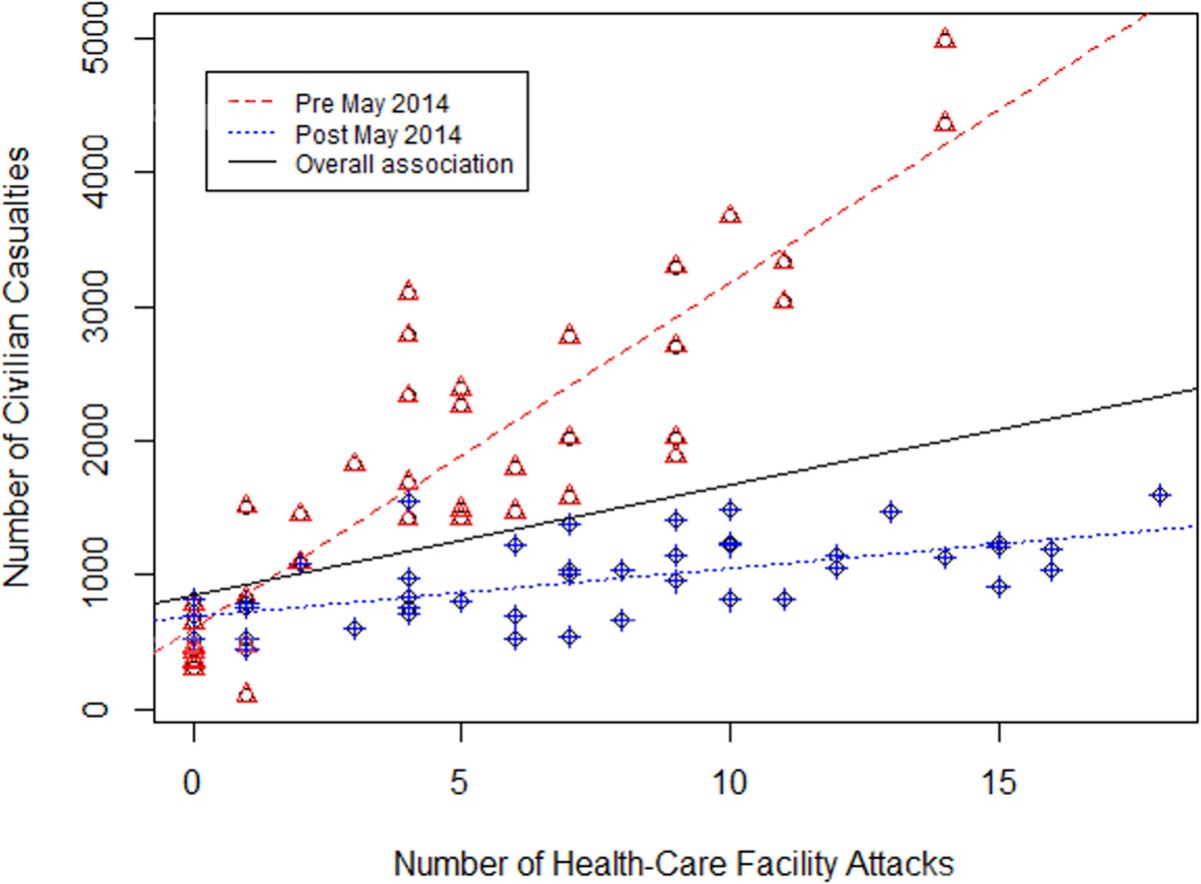
The association of civilian casualties and attacks on healthcare facilities pre-post effect modification of May 2014.

To assess whether the visual divergence between monthly civilian casualties and attacks on healthcare facilities post-May 2014 was statistically significant [Fig 1], we utilized Spearman’s correlation to compare monthly civilian casualties to attacks on healthcare facilities, with the dataset divided at May 2014. Overall, there was a statistically significant (*p<*0.001) correlation between monthly healthcare facility attacks and the monthly tally of reported civilian casualties as indicated by a moderately strong Spearman’s coefficient of 0.517. This corresponded to roughly 81.93 civilian casualties (95% CI: 41.82–122.05) associated with each healthcare facility attack. After analyzing the relationship between civilian casualties and healthcare facility attacks with May 2014 as an effect modifier, the calculation produced statistically strong correlations (p<0.001) with Spearman’s coefficient of 0.860 for May 2011 to May 2014, and 0.606 for June 2014 to November 2017. This analysis highlights that there was a much stronger correlation between pre- May 2014 healthcare facility attacks and higher levels of monthly civilian casualties, than post- May 2014 healthcare facility attacks with lower levels of monthly civilian casualties. The correlation coefficients after separating between pre- and post- May 2014 attacks and their associated trend lines are illustrated in Fig 2.

Table 2 presents the estimated level of civilian casualties calculated in association with other conflict-related variables. The unadjusted model represents bivariate associations to identify the singular effect of a variable on the estimated civilian casualty count using Spearman’s testing. The adjusted model calculates the multivariate association of significant variables and their effect on the estimated civilian casualties from calculating a multiple variable hypothesis testing model. Based on the study methodology, the adjusted model of best fit included: (1) the number of facilities attacked, (2) the May 2014 time point, (3) the number of refugees, and (4) the interaction term between May 2014 and the number of facilities attacked.

**Table 2:** Unadjusted and adjusted estimated civilian casualties and 95% confidence intervals (95% CI) with associated variables.

	Unadjusted		Adjusted	
Variable	Estimated Civilian Casualties (per person)	95% CI	Estimated Civilian Casualties (per person)	95%CI
(Intercept)	1364.25	1155.65 to 1572.84	647.39	427.84 to 866.94
Numbers of healthcare facilities attacked	81.93	41.82 to 122.05	260.18	226.52 to 293.84
Months since the start of the conflict	-12.49	-3.96 to -21.03	—-	—-
Post May 2014	-847.9	-472.43 to -1223.28	438.80	-101.77 to 979.38
Numbers of Syrian refugees (per thousand)	-0.20	-0.10 to -0.29	-0.08	—0.19 to 0.02
Interaction between number of healthcare facilities attacked and post- May 2014 variable	—-	—-	-227.68	-184.55 to -270.82

Bivariate testing showed statistically significant associations between civilian casualties and each of the variables tested. With regards to the primary variable of interest, for every attack on a healthcare facility, there were on average just under 82 civilian casualties (95% CI: 41.82 to 122.05). There was a statistically significant decrease of slightly over 12 civilian deaths per months since the start of the conflict in March 2011 per hospital attack. Likewise, there were an estimated 847.90 fewer civilian deaths per month after May 2014 when compared to pre-May 2014 (95%CI: -472.43 to -1223.28). Finally, there was a decrease of 0.20 civilian deaths for every thousand refugees, equal to one fewer death overall for every 5000 refugees that left the country after May 2014 as compared to before this time point.

Recognizing the likelihood of confounding in the bivariate relationship, the multivariable hypothesis testing model was used to calculate the strength of the association between civilian casualties and the primary exposure of healthcare facility attacks, adjusting for the other available variables to find a model of best fit. The adjusted association also includes the effect modification term to distinguish between pre- and post-May 2014, as shown in Fig 2, and healthcare facility attacks based on assumptions described in the methods section. After including all significant variables, the number of civilian casualties associated with each healthcare facility attack increased from 81.93 civilian casualties (95% CI: 41.82 to 122.05) to 260.18 civilian casualties (95% CI: 226.52 to 293.84). This shows that for each individual attack on a healthcare facility, there is a corresponding number of 260 reported civilian casualties (95%CI: 226.52 to 293.84) even when accounting for emigration of refugees and systematic changes in the conflict post May 2014. The variable of months since the start of conflict was not included in the final model, as it was consistently shown to give a worse overall model based on the methodology used to find the multivariable model of best fit. While the variable for the number of refugees (per thousand) fleeing Syria lost statistical significance, it remained in the model as it was consistently shown to strengthen the overall model fit. Likewise, the variables for pre-/post- May 2014 was no longer significant after adjustment, but it was kept in as the effect modification term for the post—May 2014 time period. The variable of healthcare facility attacks was significant with a decrease of 227.68 civilian casualties (95%CI: 184.55 to 270.82) corresponding to each healthcare facility attack post May 2014 as compared to before this time point. This indicates that while attacks on healthcare facilities continue to contribute to an ongoing increase in overall reported civilian deaths, the scale at which this increase is happening, at least those related to healthcare facility attacks, is slightly lower after May 2014.

Based on the available data used in this pilot study, it is not possible to accurately determine whether this means that facilities were ‘safer’ after May 2014, if they were simply used less thus lowering the denominator from which to derive casualties, whether the dwindling number of active facilities were attacked in quick sequence thereby diminishing the denominator of potential deaths, or due to changes in reporting patterns. With the adjusted model producing an r^2^ value of 0.8014, it can be concluded that approximately 80% of the variation seen in the model for civilian casualties can be explained with the associated variables.

## Discussion

To our knowledge, this is the first study to utilize publicly available, online data to explore the relationship between attacks on healthcare facilities and civilian casualties during an active conflict. The descriptive analysis highlights that documented attacks on healthcare facilities can be useful to understand the extent of violence against civilians in conflict. From Fig 1, there were upward trends in civilian casualties and attacks on healthcare facilities early in the conflict that diverged later in the conflict. As the war progressed, specifically from May 2014 onwards, attacks on healthcare facilities continued to increase erratically while the number of civilian casualties per month plateaued. The two indicators of violence, civilian casualties and attacks on healthcare facilities, provide different narratives on the level of violence committed against civilians in the latter period of the Syrian Civil War. Using civilian casualties as the only indicator to assess violence against civilians would suggest that Syria is becoming less dangerous for civilians, which according to attacks on healthcare facilities, might be misleading.

Fig 2 analyzes the discrepancy between pre- and post- May 2014 attacks, and civilian casualties. From March 2011 to May 2014, there was a stronger correlation between healthcare facility attacks pre-May 2014 and higher levels of civilian casualties than post-May 2014 healthcare facility attacks and lower level of civilian casualties. This discrepancy is also statistically illustrated in Table 2, and seen in the multivariable linear regression model by the effect modification term that showed a significant continual decrease of 227 civilian casualties for each healthcare attack after May 2014. This implies that the attacks on healthcare facilities are becoming less dangerous for civilians, at least in terms of their immediate mortality. However, this correlation may suggest a more deeply concerning undercurrent as civilians, witnessing increasing attacks on the few remaining facilities may eschew care even when needed, potentially leading to greater morbidity and suffering. Likewise, these healthcare facilities are one of the primary locations recording civilian casualties, and so a decreased usage would potentially mask what could be greater increase in civilian deaths. This highlights that as the Syrian conflict continued; using only civilian casualties may not be the most accurate indicator of violence against civilians. In addition, the increased attacks on healthcare facilities that are masked in the number of civilian casualties post-May 2014 suggest a more violent strategy in prevailing warring tactics.

While it is clear from the figures that civilian casualty numbers continue to increase over time since the start of the conflict, the use of months since the start of the conflict proved to not be as significant a statistical variable as the pre-/post- May 2014 variable. Mathematically, this likely results from their high collinearity, as both variables are measures of time and the better model resulting from the May 2014 moment suggests the importance of it as a critical period in the conflict. The increase in civilian healthcare facility attacks after May 2014 may be explained by political events and the introduction of new weaponry. On June 3^rd^ 2014, President Bashar Al-Assad won his third seven-year term as President of the Syrian Arab Republic and was sworn into presidency on July 16, 2014 (S2 Table). Within the next few months, the United States intervened into the Syrian conflict on September 22^nd^, 2014 to lead an anti-ISIL coalition by conducting bombing raids and escalating the conflict to a new level (S2 Table).

The Assad regime has also been well known for its systematic use of siege warfare. Over the past eight years, the Syrian government has enforced sieges on densely populated areas by depriving civilians of essential services, such as restricting access to water or humanitarian assistance, and by destroying infrastructure through aerial and on-the-ground attacks. After forcing the opposition to surrender, mass displacement of civilians is followed by government forces categorizing the evicted populations based on civilian allegiance and repopulating the re-controlled area with civilians loyal to the regime.[26] This strategy has been coined by the United Nations as a “surrender or starve” strategy which has occurred in Eastern Ghouta, Rif Damascus, Aleppo and Homs. As an integral part of the siege strategy, attacks on healthcare facilities have been linked to the sieges, specifically that of Eastern Ghouta starting around July 2013 onwards.[27]

The overall decrease in civilian casualties may be a result of how civilians, NGOs and unarmed, non-state groups have learned to adapt to the changing demands of the conflict environment. For healthcare facilities specifically, hospitals have been relocated and fortified underground to withstand aerial attacks through the construction of sacrificial floors and shatter proof windows. [28] Security procedures in hospitals have also been improved, such as ensuring the provision of personal protective equipment to healthcare personnel. Civilians have also developed coping mechanisms, such as avoiding hospitals and resorting to various types of second best alternatives from black market medical supplies or improved home expedients [29,30].

While the significant exodus of over 5.6 million Syrian refugees would seem to diminish the available population vulnerable to government attacks as seen in the unadjusted model, the effect of the exodus did not significantly alter the trend. This suggests that the vulnerability for remaining civilians remains high, highlighting the critical importance of healthcare facilities as a focal point for civilian functions.

Although the study’s multivariable linear regression model found that each healthcare facility attack corresponded to an additional 260 civilian deaths, the relationship between the two may be more complex. Elamein et al reported that 261 civilians were directly killed from the 402 incidents of violence against healthcare facilities from early November 2015 to December 2016 using their MVH reporting tool. [31] Haar et al reported that there were 90 incidents on healthcare infrastructure in 2016, the average number of deaths was 3.3 per incident, including both healthcare personnel and patients.[14] The discrepancy between these numbers highlights the complexities of trying to model aspects of conflict where multiple sources cite differing numbers. The large difference seen in our model that analyzes civilian casualties per healthcare facility attack generally and the literature that tallies civilian casualties that are directly caused by healthcare facility attacks may be due to the fact that healthcare facility attacks may occur in bombing raids that target other aspects of vital civilian infrastructure. The study also highlights that the long-term damage imposed by an attack on healthcare facility to the nation’s health system is not fully captured by the immediate casualties that result from an incident.

This research suggests that data regarding attacks on healthcare infrastructure can be utilized to augment civilian casualty data and to better understand violent conflict in the Syrian Civil War. This data may play an important role in situations when casualty counts are difficult to obtain, potentially in other conflicts as well. Compared to civilian casualties, attacks on healthcare facilities are easier to track as the damage sustained by a stationary structure leaves permanent marks that can be documented locally or remotely through satellite imagery, for an unlimited period of time. An approach looking at infrastructure attacks resolves the difficulties involved when identifying a civilian casualty that is subject to movement, burial, or decomposition, and where the reason of death is hard to determine without an autopsy. With rebel groups involved in the Syrian conflict, there is an added difficulty in distinguishing between civilian and combatant deaths. The sheer number of attacks on healthcare facilities has profound implications both in the short-term trends of emergent injuries and chronic disease, and long-lasting health consequences of a post-conflict nation with a crippled healthcare infrastructure.

This study presents findings from data presented in publicly available online databases. Open source databases are more prevalent now than at any time in the past. We highlight that there are increasing opportunities to utilize this rapidly growing medium for more formal analysis in conflict settings. The statistical analyses used in this study also illustrate that open source data can be a valuable tool in assessing conflict-related factors associated with civilian casualties. Three open source pieces of information (e.g. number of refugees, months of conflict, and healthcare facility attacks) were able to explain roughly 80% of the variation seen in civilian casualties determined by the r^2^ value. This highlights the importance of multiple dimensions of conflict to better understand the Syrian conflict dynamics and civilian violence.

### Limitations

There are significant limitations in this research study. Civilian casualty tallies are disparate and are difficult to ascertain in an active conflict. Our study uses only VDC’s database of casualties whose authors note their challenges in accessing and retrieving casualty tallies in government-controlled areas. The methodology used by VDC may subject our analysis to only represent a partial account of Syria’s conflict-related mortality as reported civilian casualties are highly localized in non-government controlled areas. However, the relative consistency in reported patterns of civilian casualty trends between VDC and other casualty databases suggest that VDC data can be used to compare monthly casualty variations. The VDC has also been regarded as the main civilian casualty database for Syrian conflict in the literature. Additionally, the reported monthly civilian casualty data trends alone could result in two conclusions: (1) the civilian casualty trend is accurate and civilian casualty rate are decreasing in the Syrian war, or (2) the continued war has impacted mortality data collection significantly where trends are inaccurate. Either way, the inability to come to a conclusion about the reported data on civilian casualties and actual mortality highlights the importance of using another indicator of conflict, namely healthcare facility attacks, to complement our understanding of violence against civilians in Syria.

Different organizations also use different methodologies which may limit direct comparisons of datasets. For instance, PHR does not publish all recorded incidents of attacks on healthcare facilities due to their stringent verification process [32]. Similarly, the VDC and UNHCR use a multi-stage verification process that may underreport total casualties and refugee counts. Due to the heterogeneity of the datasets, the investigators are unable to conduct more detailed epidemiological or statistical analysis. We highlight a visual analysis and modest statistical testing but acknowledge that this data and the corresponding analysis are limited.

Additionally, particularly in a violent and dynamic context, data extracted from the databases are subject to change as more information becomes available. For instance, the VDC updates their casualty counts daily. For this study, we utilized counts that were accurate at the time of writing.

We acknowledge a possible risk that presenting data on attacks on civilian infrastructure to fully understand violence in a region may be misinterpreted as presenting these types of attacks as inevitable. We assert that this research should serve to highlight that attacks on hospitals have a hidden cost, which is not always well described by traditional casualty data. However, these costs must be understood to gain an informed perspective on how to help affected communities and plan for their future in a post-conflict environment. This avenue of study, rather than legitimizing these incidents which are explicitly and clearly prohibited under IHL, serves to present the human cost of war, in both the short and long term.

## Conclusion

This study highlights the added value of including indicators of violence other than civilian casualties to provide a more nuanced narrative around the human costs of war. In conflict settings, damage to social infrastructure can provide insight to the warring tactics of armed forces and violence perpetrated against civilians. With the introduction of recent technological advances, investigators are offered an opportunity for a more comprehensive understanding of violence from data on healthcare facilities in conflict that may not be illustrated in civilian casualty reports.

This information is useful to better understand the dynamics of the Syrian Civil War, inform policy-makers to implement effective programs for civilian communities, and increase awareness of both the short and long-term health impacts in conflict settings. Examples include incorporating attacks on healthcare facilities as an indicator for prediction casualty models, or estimating the number of indirect deaths due to healthcare destruction. The goal of this article is to highlight the ability for open-source data to be used as a tool to assess conflict-related factors.

The presence of multiple datasets online that have not been included in this study suggest that there is a need for better reporting to improve data quality and clarity. For instance, additional variables and mixed-effect modeling could be conducted to determine whether the patterns of civilian violence from hospitals, schools, and places of worship are significant. With the increasing use of social media and access to real-time surveillance data, future researchers may be able to use other open-source data for conflict forecasting and early warning systems. With an increasing number of organizations tracking civilian casualty and healthcare facility attacks, a multiple systems estimation approach can be adopted to include multiple data sources to reconstruct mortality and violent profiles in other conflicts. For future analysis, organizations that collect and publish data may consider strengthening transparency on their respective methodology by presenting data in formats that may be easier for analysis (i.e. tables rather than individual incident reports), and working in collaboration with epidemiologists and statisticians for strengthening data collection.

As all open-source data were extracted from NGOs, there is also a need for governments to play a role in data collection. Following the adoption of the UN Security Council’s Resolution 2286 in 2016, the condemnation of attacks on healthcare infrastructure requires an active monitoring and surveillance system [33]. In the future, access to mobile and satellite technology will likely heighten the ability to accurately document attacks on civilian infrastructure and further analyses, such as those employing GIS methodologies, and will likely add value to our initial inquiry.

## Supporting information

S1 Table Monthly number of attacks on healthcare facilities, civilian casualty data and Syrian refugee tally extracted from selected open-source databases (see Table 1). (XLSX)

S2 Table Timeline of significant events in the Syrian Civil War from March 2011 to November 2017 (see Fig 1). (PDF)
