# Supplementary material for: Attacks on healthcare facilities as an indicator of violence against civilians in Syria: An exploratory analysis of open-source data
Source: PLoS One. 2019 Jun 10;14(6):e0217905. doi: 10.1371/journal.pone.0217905 (PMC6557482; doi:10.1371/journal.pone.0217905)
Supplement: S2 Table — (PDF) [file pone.0217905.s002.pdf]

S2 Table: Timeline of Significant Events in the Syrian Civil War from March 2011 to November 2017 (See Fig 1)

Category of Violence Legend

CF: Ceasefires/De-Escalation Zones/Safe-Zones

MP: Mass Protests

CW: Chemical Weapons

NP: National Politics

II: International Intervention

NW: New Weaponry Used

Date	Months since Mar. 2011	Date	Event	Category of Violence*
Mar. 2011	0	Mar. 15	"Day of Rage" as hundred stage protests in Syria for arrests of several youth in Daraa for writing graffiti to call for the downfall of the regime	MP
Apr. 2011	1	Apr. 29	United States imposes sanctions on top Syrian officials	II
May 2011	2	May 10	EU imposes sanctions on 13 of Syria's top officials	II
Jun. 2017	3	Jun. 17	Tens of thousands protest in Daraa, Deir al-Zor, Homs, Kiswa and Hama leading to arrest of 2,000 civilians and 130 civilians deaths	MP
Jul. 2011	4	Jul. 3	Assad deploys troops to areas of massive protests and open fire and conduct mass arrest	MP
Jan. 2012	10	Jan. 13	Weekly Friday protests occurred, with tens of thousands protesting in Homs, Hama, Idlib, Aleppo and Damascus.	MP
Feb. 2012	11	Feb. 10<br>Feb. 27	Mass protests spread across Syria in Aleppo, Hama, Idlib, Daraa and Damascus<br>European Union announces sanctions against Syria	MP<br>II
Mar. 2012	12	Mar. 23	Fighting between the FSA and Syrian army and weekly Friday protests continue in Damascus, Daraa, Qamishli, Hasakah, the Idlib province, Aleppo, Homs, and the Hama province	MP
Apr. 2012	13	Apr. 12	Kofi Annan sponsored ceasefire goes into effect	CF
May 2012	14	May 7	Parliamentary elections took place but boycotted by opposition forces. Vast majority of the seats given to Assad and his allies.	NP
Jul. 2012	16	Jul. 23<br>Jul. 24	EU strengthened arms embargo against Syria and toughened sanctions<br>Syrian armed forces battling insurgents start using fighter jets in Damascus	II<br>NW
Aug. 2012	17	Aug. 2<br>Aug. 10<br><br>Aug. 22	Kofi Annan resigns from the UN<br>Upsurge in violence sends thousands of Syrians fleeing to neighboring countries. 146,000 Syrians have registered as refugees since March 2011<br>Gunfire and shelling rocked Damascus and Aleppo	II<br>NW

Oct. 2012	19	Oct. 14	Government forces accused of using Russian-made cluster bombs in Syria	NW
Dec. 2012	21	Dec. 23	First allegation of chemical weapons being reported in Homs	CW
Mar. 2013	24	Mar. 19<br>Mar. 24	A gas attack kills 26 people in Khan al-assal<br>Syrian opposition activists report Syrian forces use chemical weapons in Damascus: phosphorus bombs	CW
Apr. 2013	25	----	Syrian Observatory for Human Rights (SOHR) said two gas bombs dropped in Aleppo. A helicopter dropped canisters of chemical weapons in Sarqeb township.	CW
Aug. 2013	29	Aug. 21	A sarin gas attack kills hundreds in Damascus	CW
Sep. 2013	30	Sep. 10	Syria signs the Chemical Weapons Convention (CWC)	II
Oct. 2013	31	----	Syria destroys its chemical weapons production<br>Refugees registered with UNHCR tops 2 million	NP
Jun. 2014	39	Jun. 3<br><br>Jun. 23	Asaad wins presidential election with 88.7% of the vote for a third seven-year term as President<br>OPCW has announced the destruction and removal of all Syria chemical weapon material	NP
Sep. 2014	42	Sep. 22	US leads an anti-ISIL coalition through air and missile strikes	II
Feb. 2015	47	Feb. 22	United Nations Security Council adopts Resolution 2139 demanding all parties stop indiscriminate attacks in populated areas	II
Mar 2015	48	Mar. 6	United Nations Security Council adopts Resolution 2209 condemning use of chlorine as weapon in Syria's civil war	II
Aug. 2015	53	Aug. 7	United Nations Security Council new investigation to reports that chlorine gas has been used: OPCW-UN Joint Investigative Mechanisms	II
Sep. 2015	54	Sep. 24	Zabadini Ceasefire Agreement between Free Syrian Army and Syrian Armed Forces	CF
Feb. 2016	59	Feb. 27 to early Jul.	Cessation of Hostilities I by United States and Russia	CF
Sep. 2016	66	Sep. 7<br>Sep. 9 – Sep. 19	Allegations Toxic Chemical were used in Aleppo<br>Cessation of Hostilities II by US and Russia	CW<br>CF
Dec. 2016	69	Dec. 20 – Jan. 2	Syrian Armed Forces and Free Syrian Army achieve ceasefire at Wadi Barada	CF
Apr. 2017	73	Apr. 4	Chemical weapons were used in Syria's Idlib province	CW
May 2017	74	----	Russian Syrian Safe Zones plan comes into effect	CF

Jul. 2017	76	Jul. 7	Trump and Putin agree to a limited cease fire	CF
Aug. 2017	77	Aug. 3	Hama De-escalation Agreement	CF
Sep. 2017	78	----	De-escalation Zones agreed by Iran, Turkey, Russia	CF
